# Discovery of Highly Potent Fusion Inhibitors with Potential Pan-Coronavirus Activity That Effectively Inhibit Major COVID-19 Variants of Concern (VOCs) in Pseudovirus-Based Assays

**DOI:** 10.3390/v14010069

**Published:** 2021-12-31

**Authors:** Francesca Curreli, Shahad Ahmed, Sofia M. B. Victor, Aleksandra Drelich, Siva S. Panda, Andrea Altieri, Alexander V. Kurkin, Chien-Te K. Tseng, Christopher D. Hillyer, Asim K. Debnath

**Affiliations:** 1 Laboratory of Molecular Modeling & Drug Design, New York Blood Center, Lindsley F. Kimball Research Institute, New York, NY 10065, USA; ph.shahd@hotmail.com (S.A.); sofia.mary48@gmail.com (S.M.B.V.); 2 Department of Microbiology and Immunology, The University of Texas Medical Branch, Galveston, TX 77555, USA; aldrelic@utmb.edu (A.D.); sktseng@UTMB.EDU (C.-T.K.T.); 3 Department of Chemistry & Physics, Augusta University, Augusta, GA 30912, USA; sipanda@augusta.edu; 4 EDASA Scientific, Scientific Campus, Moscow State University, Leninskie Gory Bld. 75, 77-101b, 119992 Moscow, Russia; aaltieri@edasascientific.com (A.A.); kurkin@edasascientific.com (A.V.K.); 5 Center of Biodefense and Emerging Disease, The University of Texas Medical Branch, Galveston, TX 77555, USA; 6 New York Blood Center, New York, NY 10065, USA; CHillyer@nybc.org

**Keywords:** severe acute respiratory syndrome (SARS), SARS-CoV, SARS-CoV-2, middle east respiratory syndrome (MERS), MERS-CoV, COVID-19, pan-coronavirus, fusion inhibitor

## Abstract

We report the discovery of several highly potent small molecules with low-nM potency against severe acute respiratory syndrome coronavirus (SARS-CoV; lowest half-maximal inhibitory concentration (IC_50_: 13 nM), SARS-CoV-2 (IC_50_: 23 nM), and Middle East respiratory syndrome coronavirus (MERS-CoV; IC_50_: 76 nM) in pseudovirus-based assays with excellent selectivity index (SI) values (>5000), demonstrating potential pan-coronavirus inhibitory activities. Some compounds showed 100% inhibition against the cytopathic effects (CPE; IC_100_) of an authentic SARS-CoV-2 (US_WA-1/2020) variant at 1.25 µM. The most active inhibitors also potently inhibited variants of concern (VOCs), including the UK (B.1.1.7) and South African (B.1.351) variants and the Delta variant (B.1.617.2) originally identified in India in pseudovirus-based assay. Surface plasmon resonance (SPR) analysis with one potent inhibitor confirmed that it binds to the prefusion SARS-CoV-2 spike protein trimer. These small-molecule inhibitors prevented virus-mediated cell–cell fusion. The absorption, distribution, metabolism, and excretion (ADME) data for one of the most active inhibitors, NBCoV1, demonstrated drug-like properties. An in vivo pharmacokinetics (PK) study of NBCoV1 in rats demonstrated an excellent half-life (t_1/2_) of 11.3 h, a mean resident time (MRT) of 14.2 h, and oral bioavailability. We expect these lead inhibitors to facilitate the further development of preclinical and clinical candidates.

## 1. Introduction

The outbreak of coronavirus disease 2019 (COVID-19), caused by the novel coronavirus (CoV) severe acute respiratory syndrome coronavirus 2 (SARS-CoV-2) and first reported in December 2019 in Wuhan [1], China, has led to massive human suffering, death, and economic devastation worldwide. Due to the exceptional ingenuity of academics and pharmaceutical companies, several highly effective vaccines have been developed against SARS-CoV-2 in record time, including one vaccine (Pfizer-BioNTech) that recently obtained U.S. Food and Drug Administration (FDA) approval and two vaccines (Moderna and Janssen) that have received emergency use authorization. Other COVID-19 vaccines recommended by WHO and several other countries are Covishield by Oxford/Astra-Zeneca, UK, CoronaVac from Sinovac, China, Covaxin by Bharat Biotec, India. Despite these breakthroughs in vaccine development, effective vaccines may not reach all individuals on a global scale. In addition, vaccine hesitancy is proving to be a major roadblock to achieving a satisfactory vaccination rate at the population level in many countries [2,3,4]. According to the U.S. Census Bureau’s Household Pulse Survey, up to 32% of the U.S. population is reluctant to receive the SARS-CoV-2 vaccine. Furthermore, “breakthrough” SARS-CoV-2 infections have been reported among fully vaccinated individuals [5,6]. Therefore, the development of novel drugs able to treat or prevent SARS-CoV-2 infection remains urgently necessary. SARS-CoV-2 is a positive-sense, enveloped, single-stranded RNA virus that belongs to the family Coronaviridae, along with two other CoVs that have been responsible for major outbreaks in recent years: SARS-CoV, which originated in China in 2003, and the Middle East respiratory syndrome CoV (MERS-CoV), which originated in Saudi Arabia in 2012. Although prior outbreaks were severe, they have been eclipsed by the current COVID-19 pandemic. According to data from the Johns Hopkins Coronavirus Resource Center, as of 10 September 2021, more than 223 million cases of COVID-19 and 4.6 million deaths due to COVID-19 have been reported globally. In the U.S., over 42 million cases and more than 655,000 deaths have been reported.

Although the repurposing of one broad-spectrum antiviral drug, remdesivir, was approved by the FDA for emergency use against COVID-19, remdesivir has shown limited efficacy in most clinical settings. Several monoclonal antibody (mAb)-based therapies, including those developed by Regeneron Pharmaceuticals (REGEN-COV) and Eli Lilly (bamlanivimab and etesevimab together), have also been authorized by the FDA for emergency use against COVID-19; however, their high costs and limited accessibility are prohibitive limitations for many global regions. Furthermore, most of the approved vaccines and antibody-based therapies have shown a substantial loss of potency against the SARS-CoV-2 variants that emerged in the UK (Alpha variant; B.1.1.7), South Africa (Beta variant; B.1.351), and Brazil (Gamma variant; P.1) and are now spreading across the globe [7,8,9,10]. Recently, the Delta variant (B.1.617.2) [6,11,12], which was first identified in India, has spread like wildfire. Although vaccines are critical for preventing infection and severe illness, therapeutic drugs play crucial roles in combating the disease among infected individuals. Therapeutic drugs may shorten symptomatic disease or prevent serious illness, hospitalization, or death in patients with SARS-CoV-2 infection. Currently, no FDA-approved drugs target the host cell entry or fusion mechanisms of CoVs, and the development of highly potent, novel drugs with pan-CoV activity and minimal toxicity remains urgently necessary. Currently, 245 antivirals against COVID-19 are under development (https://www.bio.org/policy/human-health/vaccines-biodefense/coronavirus/pipeline-tracker; accessed on 16 April 2021).

Similar to all enveloped viruses, CoVs begin their life cycle with entry into a host cell, which is initiated by the trimeric spike (S) surface protein [13,14]. The S protein is cleaved into S1 and S2 subunits by furin-like proteases after binding with the host cell. The S1 subunit attaches to a host cell receptor through its receptor-binding domain (RBD). The receptor for both SARS-CoV and SARS-CoV-2 is angiotensin-converting enzyme 2 (ACE2) [15,16], whereas the receptor for MERS-CoV is dipeptidyl peptidase 4 (DPP4; also termed CD26) [17]. After the S1 subunit binds a receptor, the fusion protein (FP) of the S2 subunit inserts into the cell membrane, triggering the heptad repeat region 1 (HR1) domain of the S2 subunit to form a coiled-coil trimer. The HR2 domain of the S2 subunit then binds to a hydrophobic groove in the HR1 trimer in an antiparallel manner, creating a six-helix bundle (6-HB) structure. This process brings the viral membrane and the host cell membrane into close apposition, facilitating virus–cell fusion [18], which is a critical step for viral entry into host cells. The fusion mechanism utilized by the S protein of CoVs, including MERS-CoV, resembles those used by other viral Class I membrane fusion proteins, such as those encoded by the influenza virus, human immunodeficiency virus (HIV), and Ebola virus [13,19]. However, some distinctions exist, including the larger sizes, double cleavage sites, and long 6-HB structures of the CoV S proteins [13].

Due to its exposure on the surface of the mature virus particle, the S protein is the primary target for neutralizing antibodies and vaccines. Both the S1 subunit, particularly the RBD domain, and the S2 subunit, especially the HR1 domain, have been targeted for novel drug design [20,21,22,23,24,25,26,27,28]. However, the RBD of the S1 domain is not well-conserved among CoVs [29]. Therefore, anti-S1 domain antibodies that neutralize SARS-CoV show poor cross-reactivity with SARS-CoV-2 [30,31]. In addition, several mutations have been reported in the RBD domain of SARS-CoV-2 [32,33], some of which reduce the efficacy of the antibodies induced by the currently available vaccines [34,35]. Therefore, the RBD may not be an ideal target for the development of novel pan-CoV inhibitors.

By contrast, the membrane fusion domains located in the S2 subunit are generally well-conserved and may serve as ideal targets for novel small-molecule and peptide-based pan-coronavirus inhibitors. Drugs that target the most conserved sites among CoVs will provide better broad-spectrum (pan-CoV) antiviral activity [29] and are likely to be critically important for addressing new viral pandemics that emerge in the future.

We report the identification and characterization of a series of inhibitors that show highly potent pan-CoV activity against SARS-CoV, SARS-CoV-2, and MERS-CoV. The most active inhibitors also potently inhibited mutants created in the laboratory that mimic the currently circulating variants B.1.1.7 UK (Alpha), B.1.351 RSA (Beta), and B.1.617.2 India (Delta). The lead inhibitors are expected to facilitate the further development of preclinical and clinical candidates.

## 2. Materials and Methods

### 2.1. Cells and Plasmids

MRC-5, A549, HT-1080, HeLa, HEK293T, and HEK293T/17 cells were purchased from American Type Culture Collection (ATCC; Manassas, VA, USA). Human lung carcinoma (A549) cells expressing human ACE2 (HA FLAG; A549/ACE2 cells, Catalog No. NR-53522) were obtained from BEI Resources, The National Institute of Allergy and Infectious Diseases (NIAID), National Institutes of Health (NIH). Human T-cell lymphoma Jurkat (E6-1) cells were obtained through the NIH AIDS Reagent Program (ARP). HuH-7 (JCRB0403) cells were obtained from JCRB Cell Bank (Osaka, Japan). HT1080/ACE2 (human fibrosarcoma) cells, 293T/ACE2 cells, and two plasmids, pNL4-3∆Env-NanoLuc and pSARS-CoV-2-S_Δ19_, were kindly provided by Dr. P. Bieniasz of Rockefeller University [36]. The pSV-A-MLV-Env (envelope) expression vector [37,38] and the Env-deleted proviral backbone plasmid, pNL4-3.Luc.R-E-DNA [39,40] was obtained through the NIH ARP. Two plasmids, pSARS-CoV, and pMERS-CoV, were kindly provided by Dr. L. Du of the New York Blood Center (NYBC). The expression vector containing the SARS-CoV-2 wild-type (WT) full spike gene from Wuhan-Hu-1 isolate (pUNO1-SARS-S) was purchased from InvivoGen (San Diego, CA, USA). The pFB-Luc plasmid vector was purchased from Agilent Technologies (Santa Clara, CA, USA).

### 2.2. Small Molecules

We screened a set of thirteen compounds of which nine 3-(5-((4-oxo-3-phenethyl-2-thioxothiazolidin-5-ylidene)methyl)furan-2-yl)benzoic acids were from our stock (NYBC). The synthesis, purification, and analytical characterization details are available in a prior publication [41]. We also purchased NBCoV1 from Sigma-Aldrich (St. Louis, MO, USA) in large quantities, followed by thorough purification and characterization by our group (compounds purity is >95%; details are in the Supporting Information). We purchased one control analog, lacking the COOH group, 5-((5-(4-chlorophenyl)furan-2-yl)methylene)-3-phenethyl-2-thioxothiazolidin-4-one (NBCoV15), in addition to NBCoV17, NBCoV28, and NBCoV34, from ChemBridge Corporation (San Diego, CA, USA). These compounds have diverse substitutions compared to NBCoV1 and expected to provide a comprehensive structure activity relationship (SAR). All purchased compounds had purity > 95%, and analysis details are reported in the Supporting Materials.

### 2.3. Pseudoviruses Preparation

Pseudoviruses capable of single-cycle infection were prepared using FuGENE HD (Promega, Madison, WI, USA), as previously described [27]. To obtain the SARS-CoV-2, SARS-CoV, and MERS-CoV pseudoviruses, HEK-293T/17 cells were transfected with the HIV-1 Env-deleted proviral backbone plasmid, pNL4-3∆Env-NanoLuc DNA and the pSARS-CoV-2-S_Δ19_ [36], pSARS-CoV, and pMERS-CoV Env plasmids, respectively. To generate the A-MLV pseudovirus, cells were transfected with the Env-deleted proviral backbone plasmid pNL4-3.Luc.R-E-DNA and the pSV-A-MLV-Env expression vector. Pseudovirus-containing supernatants were collected 2 days after transfection, filtered, tittered, and stored in aliquots at −80 °C. Pseudovirus titers were assessed to identify the 50% tissue culture infectious dose (TCID_50_) by infecting the different cell types. All the assays were performed in 96-well plates using 100 μL aliquots of serial 2-fold dilutions of pseudoviruses. For the titers in HT1080/ACE2 cells, 2 × 10^4^ cells were added to 100 μL aliquots of pseudoviruses and incubated for 24 h. For the titers in A549/ACE2 cells, 1 × 10^4^ cells were added to the pseudoviruses and incubated for 48 h. 293T/ACE2, MRC-5, and HuH-7 cells at 1 × 10^4^ cells/well were plated and incubated overnight before adding the pseudoviruses, followed by 48 h incubation. Following the incubation time, the cells were washed with PBS and lysed with 50 μL of the cell culture lysis reagent (Promega, Madison, WI, USA). For the SARS-CoV-2, SARS-CoV, and MERS-CoV titers, 25 µL of the lysates were transferred to a white plate and mixed with the same volume of Nano-Glo^®^ Luciferase reagent (Promega). For the A-MLV titers, 25 µL of the lysates were transferred to a white plate and mixed with 50 µL of luciferase assay reagent (Luciferase assay system, Promega). We immediately measured the luciferase activity with a Tecan SPARK multifunctional microplate reader (Tecan, Research Triangle Park, NC, USA). Wells producing relative luminescence unit (RLU) levels 10-fold that of the cell background were scored as positive. The TCID_50_ was calculated according to the Spearman–Karber method [42].

### 2.4. Analysis of S Protein Incorporation into the SARS-CoV-2, SARS-CoV, and MERS-CoV Pseudoviruses

To confirm the incorporation of the respective S proteins into the SARS-CoV-2, SARS-CoV, and MERS-CoV pseudoviruses, a 2 mL volume of the pseudovirus-containing supernatants was subjected to ultra-centrifugation for 2 h at 40,000 rpm on a 20% sucrose cushion to concentrate the viral particles. Viral pellets were lysed and processed for protein analysis. The proteins were resolved on a NuPAGE Novex 4–12% Bis-Tris Gel (Invitrogen, Carlsbad, CA, USA). The SARS-CoV-2 and SARS-CoV viral lysates were immunodetected with a SARS S protein antibody (NB-100-56578, Novus Biological, Littleton, CO, USA). The MERS-CoV viral lysate was immunodetected with a MERS-coronavirus S protein S2 polyclonal antibody (Invitrogen, Carlsbad, CA, USA). Proteins were visualized using chemiluminescence.

### 2.5. Evaluation of ACE2 and DPP4 (CD26) Expression

The expression levels of ACE2 and DDP4 were evaluated in different cell lines using western blot analysis, and correlations were examined with the infection levels detected. Cell pellets were lysed and processed for protein analysis. For Blots 1 and 3, we loaded 50 µg of protein per sample and for Blot 2, to detect ACE2 in A549 cells, 75 µg of protein was necessary. The proteins were resolved on a NuPAGE Novex 4–12% Bis-Tris Gel. Blots 1 and 2 were immunodetected with a human anti-ACE2 mAb (AC384; Adipogen Life Sciences, San Diego, CA, USA). Blot 3 was immunodetected with a human DPP4 mAb (OTI11D7; TrueMAB™; Invitrogen). Cell lysates were also immunodetected with an antibody against the housekeeping gene β-actin (Sigma) as a loading control.

Additionally, the correlations between pseudovirus SARS-CoV-2 and SARS-CoV infection rates and ACE2 expression were analyzed by infecting different cell types with the same volume of the pseudovirus-containing supernatant. Briefly, 50 µL SARS-CoV-2 or SARS-CoV pseudoviruses were diluted with 50 µL serum-free medium and added to a 96-well cell culture plate. Cells were added as follows: HT1080/ACE2 and HT-1080 cells were added at 2 × 10^4^ cells/well and incubated for 24 h at 37 °C; A549/ACE2, A549, and HeLa cells were added at 1 × 10^4^ cells/well and incubated for 48 h; 293T/ACE2 and 293T cells were added at 1 × 10^4^ cell/well, plated the day before, infected with the same volumes of SARS-CoV-2 and SARS-CoV, and incubated for 48 h. Uninfected cells for all cell lines were used as negative controls.

MERS-CoV pseudovirus infection rates were correlated with DPP4 expression following the infection of three different cell types (MRC-5, HuH-7, and HeLa cells) using the same volume of the MERS-CoV pseudovirus-containing supernatant. Uninfected cells for all cell lines were used as negative controls.

Following incubation, cells were washed with phosphate-buffered saline (PBS), lysed with 50 µL of the cell culture lysis reagent, and 25 µL lysate was transferred to a white plate and mixed with the same volume of Nano-Glo^®^ Luciferase reagent. The luciferase activity was immediately measured with a Tecan SPARK.

### 2.6. Measurement of Antiviral Activity

The antiviral activities of the NBCoV small molecules were evaluated in a single-cycle infection assay by infecting different cell types with the SARS-CoV-2, SARS-CoV, or MERS-CoV pseudoviruses, as previously described, with minor modifications [27,43]. For all neutralization assays, cells cultured in medium with and without pseudoviruses were included as positive and negative controls, respectively.

HT1080/ACE2 cells. Briefly, in 96-well culture plates, aliquots of SARS-CoV-2 or SARS-CoV pseudovirus at a concentration of approximately 3000-times the TCID_50_/well, equal to a multiplicity of infection (MOI) of 0.1, were pre-incubated with escalating concentrations of the NBCoV small molecules for 30 min. Next, 2 × 10^4^ cells were added to each well and incubated at 37 °C. Following 24 h of incubation, the cells were washed with PBS, lysed with 50 µL lysis buffer, and 25 µL lysate was mixed with the same volume of Nano-Glo^®^ Luciferase reagent. The luciferase activity was measured immediately with the Tecan SPARK. The percent inhibition by each small molecule and the half-maximal inhibitory concentration (IC_50_) values were calculated using GraphPad Prism 9.0 software (San Diego, CA, USA).

A549/ACE2 cells. Aliquots of SARS-CoV-2 or SARS-CoV pseudovirus at a concentration of approximately 1500-times the TCID_50_/well, equal to an MOI of 0.1, were pre-incubated with escalating concentrations of the NBCoV small molecules for 30 min. Next, 1 × 10^4^ A549/ACE2 cells were added to each well and incubated at 37 °C. Following 48 h of incubation, the cells were processed as described.

293T/ACE2 cells. Briefly, 96-well plates were coated with 50 µL of poly-l-Lysine (Sigma-Aldrich, St. Louis, MO, USA) at 50 µg/mL. Following a 3 h incubation at 37 °C, the plates were washed with PBS and allowed to dry. The 293T/ACE2 cells were then plated at 1 × 10^4^/well and incubated overnight. The following day, the pseudovirus aliquots, at approximately 1500-times the TCID_50_/well, equal to an MOI of 0.1, were pretreated with graded concentrations of the NBCoV small molecules for 30 min and added to the cells. Additional experiments were performed by pre-treating the cells rather than the virus with escalating concentrations of NBCoV small molecules for 30 min before infection with the SARS-CoV-2 pseudovirus. After 48 h of incubation, the cells were processed as described above. To test the specificity of the small molecules, we evaluated their activities against the A-MLV pseudovirus, at a concentration of approximately 1500-times the TCID_50_/well, equal to an MOI of 0.1, using the infection protocol described above. Following 48 h of incubation, the cells were processed as described.

MRC-5 and HuH-7 cells. MRC-5 and HuH-7 cells at 1 × 10^4^ cells/well were plated in a 96-well cell culture plate and incubated overnight. On the following day, aliquots of the MERS-CoV pseudovirus, at a concentration of approximately 1500 times the TCID_50_/well, equal to an MOI of 0.1, were pretreated with graded concentrations of the small molecules for 30 min, then added to the cells. Following 48 h of incubation, the cells were processed as described above.

### 2.7. SARS-CoV-2 Microneutralization Assay

The standard live virus–based microneutralization assay was used [44,45,46]. Briefly, serial two-fold and duplicate dilutions of individual NBCoV small molecules were incubated with 120 plaque-forming units (PFU) of SARS-CoV-2 (US_WA-1/2020) at room temperature for 1 h before transferring into designated wells of confluent Vero E6 cells (ATCC, CRL-1586) grown in 96-well cell culture plates. Vero E6 cells cultured in medium with and without the same amount of virus were included as positive and negative controls, respectively. Additional experiments were performed by pre-treating the Vero cells rather than the virus with an escalating concentration of NBCoV small molecules for 2 h before infection with SARS-CoV-2. After incubation at 37 °C for 3 days, individual wells were observed under the microscope to determine the virus-induced cytopathic effect (CPE). The efficacy of individual drugs was expressed as the lowest concentration capable of completely preventing virus-induced CPE in 100% of the wells.

### 2.8. Evaluation of Cytotoxicity

The cytotoxicity of the NBCoV small molecules was assessed in the various cell types in parallel with the antiviral activity assay, measured using the colorimetric CellTiter 96^®^ AQueous One Solution Cell Proliferation Assay (MTS) (Promega, Madison, WI, USA), according to the manufacturer’s instructions.

HT1080/ACE2 cells. Briefly, 100 µL of the NBCoV small molecules at graded concentrations were incubated with 2 × 10^4^ HT1080/ACE2 cells/well and cultured at 37 °C. Following 24 h of incubation, the MTS reagent was added to the cells and incubated for 4 h at 37 °C. The absorbance was recorded at 490 nm. The percent cytotoxicity and the concentration for 50% cytotoxicity (CC_50_) values were calculated using the GraphPad Prism 9.0 software.

A549/ACE2 cells. For cytotoxicity assays in A549/ACE2 cells, aliquots of escalating concentrations of the small molecules were incubated with 1 × 10^4^ A549/ACE2 cells/well and cultured at 37 °C for 48 h. Cytotoxicity was measured as described.

HuH-7, MRC-5, and 293T/ACE2 cells. For cytotoxicity assays in HuH-7, MRC-5, and 293T/ACE2 cells, 1 × 10^4^ cells/well were plated in a 96-well cell culture plate and incubated overnight. The following day, aliquots of escalating concentrations of the NBCoV compounds were added to the cells and incubated at 37 °C for 48 h. Cytotoxicity was measured as described.

### 2.9. Drug Sensitivity of Spike-Mutated Pseudovirus

Amino acid substitutions or deletions were introduced into the pSARS-CoV-2-S_trunc_ expression vector by site-directed mutagenesis (Stratagene, La Jolla, CA, USA) according to the manufacturer’s instructions using the following mutagenic oligonucleotides:

SaCoV2-E484K-F: 5′-ACCCCTTGTAACGGCGTGAAAGGCTTCAACTGTACTTCCCA-3′

SaCoV2-E484K-REV: 5′-TGGGAAGTAGCAGTGAGCCTTTCAGCCGTTACAAGGGG-3′

SaCoV2-N501Y-F: 5′-TCCTACGGCTTTCAGCCCACATATGGCGTGGGTATCAGCCC-3′

SaCoV2-N501Y-REV: 5′-GGGCTGATAGCCCACGCCATATGTGGGCTGAAAGCCGTAGGA-3′

SaCoV2-D614G-F: 5′-CAGGTGGCAGTGCTGTACCAGGGCGTGAACTGTACCGAAGTG-3′

SaCoV2-D614G-REV: 5′-CACTTCGGTACAGTTCACGCCCTGGTACAGCACTGCCACCTG-3′

SaCoV2-P681H-F: 5′-CAGACACAGACAAACAGCCACAGACGGGCCAGATCTGTG-3′

SaCoV2-P681H-REV: 5′-CACAGATCTGGCCCGTCTGTGGCTGTTTGTCTGTGTCTG-3′

SaCoV2-P681R-F: 5′-CAGACACAGACAAACAGCCGCAGACGGGCCAGA TCTGTG-3′

SaCoV2-P681R-REV: 5′-CACAGATCTGGCCCGTCTGCGGCTGTTTGTCTGTGTCTG-3′

SaCoV2-D950N-F: 5′-GCCCTGGGAAAGCTGCAGAACGTGGTCAACCAGAATGCC-3′

SaCoV2-D950N-REV: 5′-GGCATTCTGGTTGACCACGTTCTGCAGCTTTCCCAGGGC-3′

SaCoV2-∆(69–70)-S: 5′-GTGACCTGGTTCCACGCCATCTCCGGCACCAATGGCACCAAG-3′

SaCoV2-∆(69–70)-REV: 5′-CTTGGTGCCATTGGTGCCGGAGATGGCGTGGAACCAGGTCAC-3′

Site mutations were verified by sequencing the entire spike gene of each construct. To obtain the SARS-CoV-2 pseudovirus carrying the amino acid substitutions, the cells were transfected with the HIV-1 Env-deleted proviral backbone plasmid pNL4-3∆Env-NanoLuc DNA and the mutant pSARS-CoV-2-S_Δ19_, as described above. Pseudoviruses were titered by infecting 293T/ACE2 cells. To measure the activity of the compounds against pseudoviruses expressing different point mutations, 293T/ACE2 cells were infected with the ENV-mutated pseudoviruses, as described above.

### 2.10. Cell-to-Cell Fusion Inhibition Assay

For the SARS-CoV-2–mediated cell-to-cell fusion assay, we used Jurkat cells transiently expressing the luciferase gene and stably expressing the SARS-CoV-2 full spike WT gene from Wuhan-Hu-1 isolate as donor cells and 293T/ACE2 cells as acceptor cells. Briefly, Jurkat cells at 2 × 10^5^ cells/mL were transfected with 1 µg/mL of SARS-CoV-2 WT expression vector using 5 µL/mL FuGENE HD, according to the manufacturer’s instructions. Following 24 h of incubation, transfected Jurkat cells were washed and selected for SARS-CoV-2 S protein expression using blasticidin at a concentration of 10 µg/mL. To rule out blasticidin resistance, untransfected Jurkat cells were exposed to the same blasticidin concentration in parallel, which resulted in complete culture depletion in approximately 14 days. The antibiotic was replaced every four days, and the selection process lasted for approximately 20 days. On the day before the assay, 293T/ACE2 cells were plated in a 96-well cell culture plate at 8 × 10^4^ cells/well. Jurkat cells were washed with PBS to remove the blasticidin, resuspended at 2 × 10^5^ cells/mL, and transfected with 1 µg/mL pFB-Luc expression plasmid DNA using 5 µL/mL FuGENE HD. Following 20 h of incubation, the Jurkat cells were washed with PBS, and aliquots of 8 × 10^4^ cells/well were incubated with escalating concentrations of the NBCoV compounds for 1 h. Finally, the Jurkat cells were transferred to wells containing 293T/ACE2 cells. 293T/ACE2 cells cultured in medium with and without transfected Jurkat cells were included as positive and negative controls, respectively. As an additional control, a set of 293T/ACE2 cells were incubated with Jurkat cells expressing the luciferase gene only (Jurkat-Luc). The plate was spun for 5 min at 1500 rpm and incubated for 4 h at 37 °C. The wells were carefully washed twice with 200 µL PBS to remove unfused Jurkat cells. Finally, the cells were lysed to immediately measure luciferase activity, and the percentage of inhibition of SARS-CoV-2–mediated cell-to-cell fusion was calculated.

### 2.11. Binding Analysis by SPR

A binding study examining two of the most active small-molecule inhibitors was performed by Profacgen, New York, NY, USA. Prior to use, the bare gold-coated (thickness: 47 nm) PlexArray Nanocapture Sensor Chip (Plexera Bioscience, Seattle, WA, USA) was prewashed with 10× PBS containing Tween 20 (PBST) for 10 min, 1× PBST for 10 min, and twice in deionized water for 10 min before being dried under a stream of nitrogen. Various concentrations of biotinylated proteins, the recombinant SARS-CoV-2 Spike His-tag protein (R&D Systems) and the recombinant SARS-CoV-2 Spike S1 (Bioss Antibodies, Woburn, MA, USA) were dissolved in water and manually printed onto the chip using Biodo bioprinting at 40% humidity via biotin-avidin conjugation. Each concentration was printed in duplicate, and each spot contained 0.2 µL sample solution. The chip was incubated in 80% humidity at 4 °C overnight, followed by rinsing with 10× PBST for 10 min, 1× PBST for 10 min, and twice with deionized water for 10 min. The chip was then blocked with 5% (*w*/*v*) non-fat milk in water overnight, followed by washing with 10× PBST for 10 min, 1× PBST for 10 min, and twice with deionized water for 10 min before being dried under a stream of nitrogen. SPR measurements were performed with PlexAray HT (Plexera Bioscience, Seattle, WA, USA). Collimated light (660 nm) passes through the coupling prism, reflects off the SPR-active gold surface, and is received by the charge-coupled device camera. Buffers and samples were injected by a non-pulsatile piston pump into the 30-µL flowcell mounted on the coupling prism. Each measurement cycle contained four steps: washing with PBST running buffer at a constant rate of 2 µL/s to obtain a stable baseline, sample injection at 5 µL/s for binding, surface washing with PBST at 2 µL/s for 300 s, and regeneration with 0.5% (*v*/*v*) H_3_PO_4_ at 2 µL/s for 300 s. All measurements were performed at 25 °C. Signal changes after binding and washing were recorded in arbitrary units (AU) as the assay values.

Selected protein-grafted regions in the SPR images were analyzed, and the average reflectivity variations in the selected areas were plotted as a function of time. Real-time binding signals were recorded and analyzed by Data Analysis Module (DAM, Plexera Bioscience, Seattle, WA, USA). Kinetic analysis was performed using BIAevaluation 4.1 software (Biacore, Inc., Uppsala, Sweden).

### 2.12. In Vitro ADME Study

Details of the in vitro absorption, distribution, metabolism, and excretion (ADME) study, and data analyses can be found in the Supporting Materials.

### 2.13. In Vivo Pharmacokinetics in Rats

We selected two of the most active inhibitors, NBCoV1 and NBCoV2, to evaluate the pharmacokinetics (PK) in rats. Rats were 11 weeks and 1 day old and weighed between 200–250 g. A total of 12 female Sprague Dawley rats (Charles River Laboratory, Wilmington, MA, USA) were implanted with a jugular vein catheter and assigned to the study following acclimation for 7 days. Rats were divided into four treatment groups consisting of three rats each. On Day 0, 10 mg/kg was administered to each animal via oral gavage for groups 1 and 3, whereas 5 mg/kg was administered to each animal via tail vein injection for groups 2 and 4. All animals underwent blood collection for plasma at 5, 15, and 30 min, 1, 2, 4, 8, and 24 h post-dosing. At 24 h post-dosing, all animals were euthanized after terminal blood collection without performing a necropsy. The study was conducted under BSL-1 safety conditions.

The concentrations of the test agent in plasma were determined using high-performance liquid chromatography with tandem mass spectrometric (HPLC-MS/MS). The test agent was isolated by liquid-liquid extraction. A partial aliquot of the supernatant was transferred to a clean 96-well collection plate, evaporated to dryness under nitrogen, and reconstituted with water. The extracted samples were analyzed using a Sciex 5500 mass spectrometer. The quantitative range of the assay was 1–2000 ng/mL.

PK parameters were calculated using PkSolver [47], and graphs were generated using PkSolver.

Ethic statement: This study was conducted in compliance with the current version of the following (1) Animal Welfare Act Regulations (9 CFR); (2) U.S. Public Health Service Office of Laboratory Animal Welfare (OLAW) Policy on Humane Care and Use of Laboratory Animals; (3) Guide for the Care and Use of Laboratory Animals (Institute of Laboratory Animal Resources, Commission on Life Sciences, National Research Council, 1996); and (4) AALAC accreditation. The study described in this report does not unnecessarily duplicate previous work. Procedures used in this study have been designed to avoid or minimize unacceptable discomfort, distress or pain to the animals.

Additional Methods: Details regarding the (1) enzyme inhibition assay, (2) fluorescence/luminescence interference test, and (3) colloidal aggregation study can be found in the Supporting Materials.

## 3. Results

### 3.1. Rationale for Screening HIV-1 Fusion Inhibitors as Possible Pan-CoV Inhibitors

The CoV S protein plays critical roles in the binding of the virus to a cellular receptor and the subsequent fusion and entry into host cells, allowing for the release of viral genetic material that is necessary for the continuation of the viral life cycle. The FP inserts into the host cell membrane and triggers the formation of a coiled-coil trimer by HR1. HR2 binds to the trimer’s hydrophobic groove in an antiparallel manner, creating a 6-HB, similar to the process reported for HIV-1 gp41-mediated fusion [48,49]. However, no sequence homology exists between the HR1 and HR2 regions of the CoV S protein and the respective HR1 and HR2 regions of HIV-1 gp41. The formation of the 6-HB facilitates the apposition of the virus and host cell membranes, allowing for the fusion process to complete.

The mechanistic similarity between the fusion processes utilized by SARS-CoV and HIV-1 led Jiang et al. to design peptide-based inhibitors with pan-CoV activity based on the HR2 domains of SARS-CoV [50] and SARS-CoV-2 [25]. Jiang et al. crystallized the postfusion hairpin structure of SARS-CoV-2 (6LXT) [25] and compared that structure with the corresponding SARS-CoV structure (1WYY) [51], which was reported in 2005. Remarkably, the postfusion hairpin structures were not only structurally similar but also shared critical salt bridges between the HR1 and HR2 regions [25]. In SARS-CoV-2, K947 of the HR1 domain forms a salt bridge with E1182 of the HR2 domain, whereas in SARS-CoV, K929 of the HR1 domain forms a salt bridge with E1163 of the HR2 domain. An examination of the MERS-CoV postfusion S protein structure showed a salt bridge in a similar position, with K1021 forming a salt bridge with E1265 [52]. We previously reported a similar salt bridge interaction in the HIV-1 gp41 hairpin structure, in which K547 of the N-terminal HR1 region interacts with D632 of the C-terminal HR2 region [53]. We also reported the design of a series of highly potent, benzoic acid–based HIV-1 gp41 fusion inhibitors, which each contain a COOH group [41].

**Figure 1:**
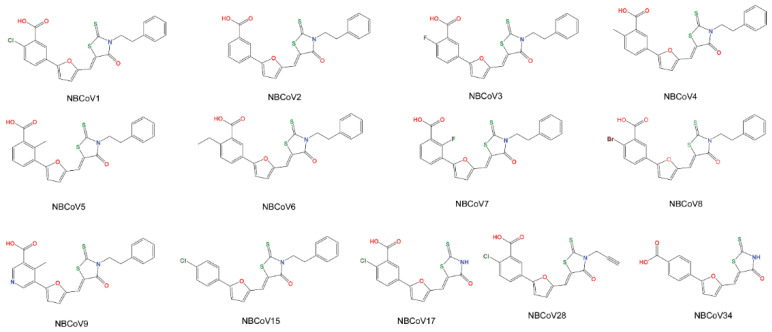
The structures of all of compounds derived from 5-((5-4-cholorophenyl)furan-2-yl)methylene)-3-phenethyl-2-thioxothiazolidin-4-one that were tested against SARS-CoV-2, SARS-CoV, and MERS-CoV.

Using computer-based docking, we found that the COOH group of these inhibitors might interrupt the hairpin structure formation by interacting with K547 and snuggly fitting into the hydrophobic grove created by the three HR1 regions of the trimer [41]. These remarkable similarities in the viral fusion mechanisms and the involvement of salt bridges in the formation of the 6-HB between HIV-1 gp41 and CoVs led us to hypothesize that this class of inhibitors may also interrupt CoV salt bridge formation by fitting into a cavity of the prefusion trimer, preventing 6-HB formation and inhibiting viral fusion with host cells. We screened a set of nine such compounds from our stock, in addition to four new analogs (NBCoV15, NBCoV17, NBCoV28, and NBCoV34) purchased commercially, against the S proteins of SARS-CoV-2, SARS-CoV, and MERS-CoV using a pseudotype antiviral assay (Figure 1). We added the new four analogs to derive a comprehensive structure–activity relationship (SAR). Recently, peptide-based pan-CoV fusion inhibitors were shown also to possess potent inhibitory activity against HIV-1, HIV-2, and SIV [54].

The selected inhibitors contain a well-known frequent hitter scaffold, ene-rhodanine, identified as pan-assay interference compounds (PAINS) [55,56,57]. However, instead of discarding these molecules as most likely promiscuous, we decided to demonstrate in pain-staking detail that this represents a privileged scaffold in the context of pan-CoV inhibition, as reported by others [58].

### 3.2. Validation of the Pseudoviruses

**Figure 2:**
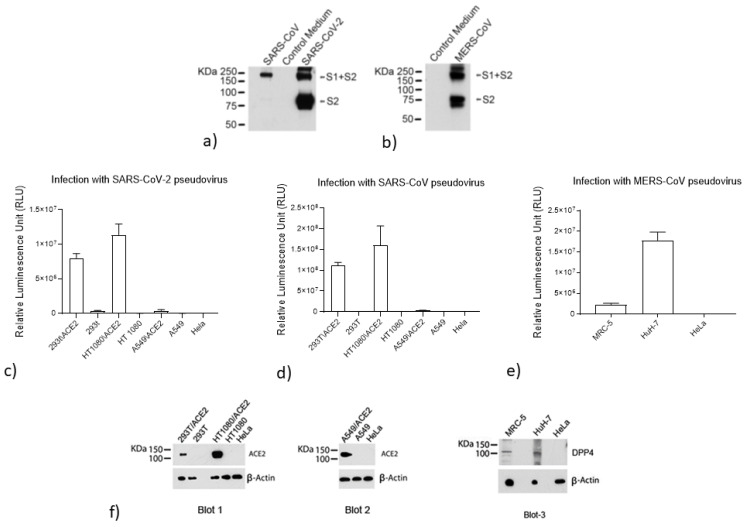
Validation of the SARS-CoV-2, SARS-CoV, and MERS-CoV pseudoviruses and ACE2 and DPP4 expression in different cell lines. (**a**) Immunoblot to validate the incorporation of the spike (S) protein in the SARS-CoV and SARS-CoV-2 pseudoviruses (**b**) Immunoblot to validate the incorporation of the spike (S) protein in the MERS-CoV pseudovirus Infection of cells expressing different levels of angiotensin-converting enzyme 2 (ACE2) with (**c**) the same amounts of SARS-CoV-2 or (**d**) SARS-CoV pseudovirus. (**e**) Infection of cells expressing different levels of dipeptidyl peptidase 4 (DPP4), with the same amounts of MERS-CoV pseudovirus. Columns represent the means ± standard deviations (*n* = 4). (**f**) Immunoblot of cell lysates to evaluate ACE2 expression (Blot 1 and Blot 2) and DPP4 expression (Blot 3). β-Actin was used as a loading control.

We prepared SARS-CoV-2, SARS-CoV, and MERS-CoV pseudoviruses capable of single-cycle infection by transfecting HEK293T/17 cells with an HIV-1 Env-deleted proviral backbone plasmid, pNL4-3∆Env-NanoLuc, together with the respective spike plasmid [36]. We then validated the incorporation of the S proteins into the respective pseudoviruses by western blot analysis. We used the SARS S protein antibody (Novus Biologicals) to detect the S2 protein in the SARS-CoV-2 and SARS-CoV pseudoviruses (Figure 2a) and a MERS-CoV S2 protein polyclonal antibody (Invitrogen) to detect the S2 protein in the MERS-CoV pseudovirus (Figure 2b). For SARS-CoV-2, we identified a specific band at 80 kDa, which identifies the S2 subdomain, and a second band at approximately 190 kDa, which corresponds to the full-length S protein (S1 + S2), as previously reported [27,43,59]. For SARS-CoV, the same antibody detected a lighter band at 80 kDa, representing the S2 subunit, and a 190 kDa band, corresponding to the full-length S protein (Figure 2a). For MERS-CoV, we identified the S2 subdomain at 75 kDa and the full-length S protein at approximately 185 kDa (Figure 2b), as previously reported [30,60]. Thus, these analyses confirmed the correct incorporation of the S proteins into their respective pseudoviruses.

We analyzed the correlation between SARS-CoV-2 and SARS-CoV pseudovirus infection levels with the expression levels of the hACE2 receptor using three different cell types that overexpress the ACE2 receptor: human kidney 293T/ACE2 cells, human fibrosarcoma HT1080/ACE2 cells, and the human lung carcinoma cells A549/ACE2. The respective parental cell types, HEK293T cells, HT1080 cells, and A549 cells, were used as controls. We also utilized HeLa cells as a control that does not express the hACE2 receptor. The cells were exposed to the same volumes of supernatant containing the respective pseudoviruses. As expected, the pseudoviruses failed to infect HeLa cells (Figure 2c,d). Similarly, we detected only low levels of SARS-CoV-2 pseudovirus infection for the parental cell lines HEK293T, HT1080, and A549 compared with the corresponding cells overexpressing the ACE2 receptor. 293T/ACE2 and HT1080/ACE2 cells supported high levels of SARS-CoV-2 infection, measured at approximately 8 × 10^6^ RLU and 1.1 × 10^7^ RLU, respectively, corresponding to 24- and 490-fold higher infection rates than were detected for the parental cell types, HEK293T and HT1080, respectively. The infection detected in A549/ACE2 cells was moderate (approximately 3.8 × 10^5^ RLU) compared with HT1080/ACE2 and 293T/ACE2 cells and approximately 13-fold higher than was detected for the parental cell type, A549. Similar results were observed for the SARS-CoV infection study, with 293T/ACE2 and HT1080/ACE2 cells supporting higher infection rates than were detected for the respective parental cell types or for A549/ACE2 cells. We confirmed these results by analyzing the expression levels of the ACE2 receptor in the different cell lines by western blot (Figure 2f). As shown in Blot 1, we found that ACE2 expression was undetectable in the parental 293T and HT1080 cell lines, whereas ACE2 overexpression was detected in HT1080/ACE2 cells. A lower amount of ACE2 was detected in 293T/ACE2 cells than in HT1080/ACE2 cells, which corresponds with the relative infection levels observed (Figure 2c,d). The reduced infection rate detected in A549/ACE2 cells suggested a lower level of ACE2 expression in these cells. To visualize ACE2 expression in Blot 2 (Figure 2f), a higher protein concentration (75 µg) was required, combined with a 2-fold antibody concentration. These data confirmed that the SARS-CoV-2 and SARS-CoV pseudoviruses infection rates were correlated with ACE2 receptor expression.

To analyze the correlation between MERS-CoV pseudovirus infection levels and DPP4 (CD26) receptor expression levels, we infected fibroblast cell lines, including the lung-derived MRC-5 cells and hepatocyte-derived carcinoma HuH-7 cells; as a control, we also infected HeLa cells that do not express the DPP4 receptor. Cells were exposed to the same volumes of supernatant containing the MERS-CoV pseudovirus. We found that HuH-7 cells supported an 8.6-fold higher level of MERS-CoV infection than MRC-5 cells, resulting in approximately 2 × 10^7^ RLU and 2.3 × 10^6^ RLU, respectively. We observed no infection of HeLa cells (Figure 2e). The expression levels of the DPP4 receptor in the two cell lines are shown in Blot 3 (Figure 2f). These data confirmed that the S proteins were correctly incorporated into their respective pseudoviruses and verified that the cellular infection with these pseudoviruses corresponded with the expression levels of the expected interacting receptors.

### 3.3. Antiviral Activity and Cytotoxicity of the NBCoV Small Molecules in a Pseudovirus Assay

We evaluated the anti-CoV activity of NBCoV small molecules by infecting the three ACE2-overexpressing cell types, 293T/ACE2, HT1080/ACE2, and A549/ACE2 cells, with aliquots of the SARS-CoV-2 pseudovirus, following pretreatment of the pseudovirus with escalating concentrations of the NBCoV small molecules for 30 min. We calculated the IC_50_ value for each NBCoV small molecule against SARS-CoV-2 pseudovirus infection, and the results are reported in Table 1. Most of the NBCoV compounds inhibited SARS-CoV-2 infection with low-nM activity. The only exceptions were NBCoV5, which had µM activity (1205 ± 240 nM, 1050 ± 252 nM, and >2000 nM in 293T/ACE2, HT1080/ACE2, and A549/ACE2 cells, respectively), and NBCoV15, which was used as a control compound because it lacks a COOH group in the phenyl ring and showed no antiviral activity, even at 2000 nM. Additionally, the compounds NBCoV17, NBCoV28, and NBCoV34 showed no antiviral activity at concentrations up to 2000 nM (the highest concentration tested). NBCoV1, NBCoV2, and NBCoV4 were the most potent compounds. The IC_50_ values calculated for NBCoV1 were in the range of 32.3 to 63.4 nM, and the selectivity index (SI; calculated as CC_50_/IC_50_) ranged from 755 to 2755; for NBCoV2, the calculated IC_50_ values were in the range of 22.8 to 58 nM, and the SI values varied from 1630 to >4000; finally, for NBCoV4 the IC_50_ values were in the range of 26 to 73 nM, and the SI values ranged from >1370 to >2096. NBCoV3 also displayed potent anti–SARS-CoV-2 activity, but the IC_50_ and SI values obtained for the three cell lines were slightly higher than those detected for NBCoV1, NBCoV2, and NBCoV4 (IC_50_: 60.1–120 nM; SI: 750 to >1563). The remaining NBCoV compounds (NBCoV6–NBCoV9) showed lower potency than NBCoV1–NBCoV4, as demonstrated by higher IC_50_ and SI values. We noticed that all compounds displayed better activity when tested in 293T/ACE2 and HT1080/ACE2 cells than when tested in A549/ACE2 cells, which were consistently associated with increased IC_50_ and SI values. Representative dose-response curves of the antiviral activity of NBCoV1 and NBCoV4 in the different cell lines are reported in Figure S1a. It has been reported that TMPRSS2 expression on target cells influences the entry of SARS-CoV-2 [61]. To verify whether there is a correlation between TMPRSS2 expression and the potency of the inhibitors, we analyzed the expression of TMPRSS2 in the cells used in our assays (Figures S2 and S3). We found that 293T/ACE2 cells expressed higher levels of TMPRSS2 respect to HT1080/ACE2 and A549/ACE2, but we could not find a direct correlation between expression levels of TMPRSS2 and potency of the inhibitors. Variable expression of TMPRSS2 in different cell lines used for measuring antiviral potency has been reported [62,63,64,65]. The cytotoxicity (CC_50_) of the small molecules was assessed in parallel with their inhibitory activities (Table 1) for use in determining the SI values. As noted, in some cases (HT1080/ACE2 and A549/ACE2 cell lines), the small molecules did not induce any apparent toxicity, even at the highest dose tested (100 µM; Table 1). As expected, when the cells (rather than the virus) were pretreated with the NBCoV compounds for 30 min before infection, no protection against SARS-CoV-2 infection was observed, even at the highest tested dose (2000 nM; Table S1). These data validate the hypothesis that these compounds target the virus rather than the cell.

To derive a SAR, we observed that NBCoV2, which does not have any para-substituents in the carboxyphenyl ring, showed the best antiviral potency against SARS-CoV-2. NBCoV1, NBCoV4, and NBCoV8, which all contain a hydrophobic substituent at the para position of the carboxyphenyl ring, also showed excellent antiviral activity. NBCoV3 has a highly electronegative fluoro atom at the para position of the carboxyphenyl ring, which corresponded with slightly reduced antiviral activity compared with NBCoV2 and NBCoV4. A bulkier hydrophobic substituent (–CH_2_CH_3_) in NBCoV6 was also associated with slightly reduced antiviral activity. Furthermore, a substituent at the ortho position in the carboxyphenyl ring was not well-tolerated, resulting in poor antiviral activity. The introduction of nitrogen in the phenyl ring (pyridine) did not improve the antiviral activity, although the solubility of this molecule may have improved. We hypothesized that the COOH groups found in these compounds might interact with key, positively charged residues in the HR1 region, interfering with the 6-HB formation; therefore, we tested an analog devoid of the COOH group (NBCoV15). As expected, NBCoV15 showed no antiviral potency, even at the highest dose tested. We extended the SAR analysis by substituting the phenylethyl group with either H in the rhodanine moiety (NBCoV17 and NBCoV34) or with a smaller hydrophobic group (prop-1-yne; NBCoV28). The position of the COOH group is also critical. When the COOH group is located at the para position of the phenyl ring (NBCoV34), the inhibitory activity is lost. These data were able to generate an insightful SAR using only a few compounds.

Although NBCoV17 and NBCoV28 both contain COOH and Cl groups at the same positions as those found in NBCoV1, they did not show any inhibitory activities, even at the highest dose tested. These data indicate that a combination of electronic and appropriately hydrophobic interactions is essential for the antiviral potency of this series of molecules. The mere presence of the ene-rhodanine moiety does not appear to be involved in the inhibitory process. NBCoV34, which features a carboxylic group at the para position and no hydrophobic group attached to the NH of the rhodanine moiety, showed no inhibitory activity. If the ene-rhodanine scaffold played any role in antiviral potency through its promiscuous nature, as has previously been suggested, NBCoV34 would be expected to display potent antiviral activity due to the lack of steric hindrance on the rhodanine scaffold, allowing for the non-specific binding of random protein targets. Based on the above observations, we only selected the most active inhibitors to test against other CoVs.

To assess whether NBCoV small molecules have pan-CoV antiviral activity, we tested them against the SARS-CoV pseudovirus (Table 2). The most potent anti–SARS-CoV compounds were NBCoV1–NBCoV4. Compounds NBCoV1–NBCoV3 had excellent IC_50_ values ranging from 13.8 to 17 nM (SI: 2265–2717) in 293T/ACE2 cells, from 19.3 to 39 nM (SI: 2282 to >5181) in HT1080/ACE2 cells, and from 98 to 157 nM (SI: >637 to >901) in A549/ACE2 cells. Compound NBCoV4 had slightly higher IC_50_ values in 293T/ACE2 cells and HT1080/ACE2 cells (IC_50_: 80 ± 2 nM and SI: 509; IC_50_: 54 ± 3.5 nM and SI: >1852, respectively) but displayed the second-best activity (IC_50_: 100 ± 13 nM) and best SI (>1000) in A549/ACE2 cells compared with NBCoV1–NBCoV3. NBCoV5 demonstrated poor activity against SARS-CoV for all three cell lines tested. Compounds NBCoV6–NBCoV9 displayed anti–SARS-CoV activity in all examined cell lines, but these compounds were less potent and displayed lower consistency than NBCoV1–NBCoV4. Representative dose-response curves of the antiviral activity of NBCoV1 and NBCoV4 in the different cell lines are reported in Figure S1b. The SAR for SARS-CoV followed a similar pattern as that observed for SARS-CoV-2, which was expected due to the high sequence and structural similarities in the S2 domain of the S protein between these viruses.

The NBCoV small molecules were also evaluated against the MERS-CoV pseudovirus by infecting HuH-7 cells and MRC-5 cells. As reported in Table 3, we found that NBCoV1–NBCoV4 were the most potent compounds against this pseudovirus in both cell lines (IC_50:_ 95–158 nM and SI > 582 in HuH-7 cells; IC_50:_ 76.5–123 nM and SI > 407 in MRC-5 cells). NBCoV9 also demonstrated noteworthy MERS-CoV inhibitory activity, with an IC_50_ value of approximately 200 nM. NBCoV5 showed no MERS-CoV inhibitory activity, even at the highest dose used in this assay (2000 nM), whereas the compounds NBCoV6–NBCoV8 inhibited MERS-CoV infection with IC_50_ values lower than 1000 nM. Representative dose-response curves of the antiviral activity of NBCoV1 and NBCoV4 are reported in Figure S1c. These findings suggest that most of the NBCoV compounds possess pan-CoV inhibitory activities. We also observed a similar SAR for NBCoVs with MERS-CoV as observed for the SARS-CoVs. To verify the specificity of the NBCoV compounds for CoVs, we evaluated their activities against amphotropic murine leukemia virus (A-MLV), which enters cells via macropinocytosis [66]. None of the NBCoV small molecules showed appreciable inhibitory activities against the control pseudovirus (IC_50_ > 783 nM; Table 4). These data suggest that the inhibitory activities of NBCoV small molecules are generally specific to CoVs; however, NBCoV1–NBCoV9 have previously demonstrated anti–HIV-1 fusion activities.

### 3.4. NBCoV Small Molecules Inhibit a Replication-Competent Authentic SARS-CoV-2 (US_WA-1/2020)

The antiviral activities of NBCoV small molecules were evaluated by exposing Vero E6 cells to a replication-competent, authentic SARS-CoV-2 (US_WA-1/2020). On the third day post-infection, the cells were observed under a microscope to evaluate the induction of a virus-induced CPE. The small-molecule efficacy was expressed as the lowest concentration capable of completely preventing the induction of any virus-induced CPE (IC_100_), and both, NBCoV1 and NBCoV2 were the most efficient compounds for preventing virus-induced CPEs, with IC_100_ values of 1.25 µM followed by NBCoV3, NBCoV4, and NBCoV9, which completely prevented virus-induced CPEs at 2.5 µM. NBCoV7 and NBCoV8 also prevented virus-induced CPEs with IC_100_ values of 5 µM, whereas NBCoV5 and NBCoV6 were unable to completely prevent virus-induced CPEs at 10 µM, which was the highest dose used in this assay (Table 5). Variation of antiviral activity in different cell lines has been reported [67]. To compare the potency of the NBCoV compounds against the pseudovirus versus the potency against the authentic virus, we calculated the IC_100_ values obtained with the pseudovirus assay (Table 5). Considering two completely different assay methods and experimental variations, for the two most active inhibitors, NBCoV1 and NBCoV2, the IC_100_ values were similar in the two assays, in fact, the values obtained with the pseudovirus assays were in the range from 0.25 µM to 1 µM and the IC_100_ obtained for the authentic virus was 1.25 µM. Moreover, when the cells were pretreated for 2 h prior to infection, the compounds did not show any protective effects against SARS-CoV-2 infection, even at the highest dose used in the assay (10 µM; Table S1). These findings support the results obtained with the single-cycle, pseudovirus-based antiviral assays.

### 3.5. NBCoV Small Molecules Neutralize the SARS-CoV-2 Variants B.1.1.7 UK (Alpha), B.1.351 RSA (Beta), and B.1.617.2 India (Delta)

Like other RNA viruses, CoVs depend on an error-prone, RNA-dependent RNA polymerase to facilitate viral replication and adaptation [68]. The emergence of major SARS-CoV-2 variants harboring multiple mutations in the spike sequence has resulted in concerns regarding increased virulence and reduced vaccine efficacy. In this study, we focused our attention on three variants that have spread globally: B.1.1.7 UK (Alpha), B.1.351 RSA (Beta), and B.1.617.2 India (Delta). We evaluated the potency of the NBCoV small molecules against these SARS-CoV-2 variants, which feature single or multiple key spike sequence mutations (B.1.1.7 UK variant: 69–70 deletion (∆69–70)/N501Y/P681H; B.1.351 RSA: E484K/N501Y/D614G; and B.1.617.2 Delta: D614G/P681R/D950N) [69,70,71,72] (Table 6). We introduced these amino acid substitutions into the pSARS-CoV-2-S_trunc_ expression vector and infected 293T/ACE2 cells with WT and mutant SARS-CoV-2 pseudoviruses in the absence or presence of NBCoV1–NBCoV4. We used NBCoV5 as a control due to its poor activity anti-CoV activity. We observed that NBCoV1 had potent antiviral activity against all mutant pseudoviruses carrying single-, double-, or triple-mutations featured in the B.1.1.7 UK, B.1.351 RSA, and B.1.617.2 Delta variants, as indicated by low IC_50_ values, which were similar to the IC_50_ values obtained for the SARS-CoV-2 WT pseudovirus (Table 6). NBCoV2 was also a highly potent inhibitor of all mutant pseudoviruses, although the IC_50_ value for the SARS-CoV-2 WT pseudovirus was lower than those detected against the mutant pseudoviruses (IC_50_ values in the range of 35–88.7 nM for all pseudovirus variants). NBCoV4 was highly potent against all of the variants but exhibited a significant increase in the IC_50_ values for the B.1.1.7 UK triple mutant variant ∆69–70/N501Y/P681H (IC_50_ of 158 nM) and the B.1.617.2 Delta single, double, and triple mutant variants (IC_50_ values of 148–239 nM). The mechanisms through which these combinations of mutations reduce the antiviral potency of NBCoV4 remains unknown; therefore, more experiments remain necessary to explain these findings. However, the compound retained appreciable antiviral activity against these mutants. NBCoV3 was slightly less efficient against all mutant pseudoviruses tested, including the WT pseudovirus. We found a higher IC_50_ when tested against the B.1.1.7 UK triple mutant variant (IC_50_ of 232 nM). Finally, NBCoV5 had poor or no activity against these variants. Representative dose-response curves of the antiviral activity of NBCoV1 and NBCoV4 against the SARS-CoV-2 mutant variants are reported in Figure S1d. These results suggest that the NBCoV small molecules maintain their potency against the three mutant SARS-CoV-2 variants tested. However, further experiments with authentic variants are needed to give more credence to the data generated here from pseudovirus-based assay.

### 3.6. Binding Affinity of the Two Most Potent Inhibitors by SPR Analysis

**Figure 3:**
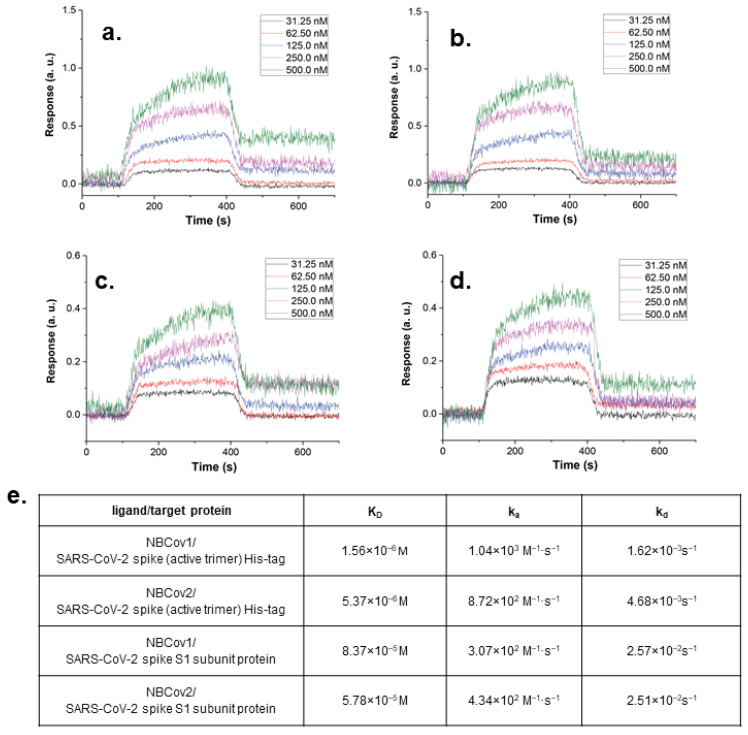
Evaluation of binding affinity of NBCoV1 and NBCoV2 to SARS-CoV-2 active trimer and SARS-CoV-2 S1 subdomain by SPR. Kinetics fitting curve (sensogram) of SARS-CoV-2 trimer to (**a**) NBCoV1; (**b**) NBCoV2. Kinetics fitting curve (sensogram) of SARS-CoV-2 S1 subdomain to (**c**) NBCoV1 and (**d**) NBCoV2. The binding affinity K_D_ and kinetic parameters k_on_ and k_off_ of (**e**) NBCoV1 and NBCoV2.

We used SPR to determine the binding affinities of the two most active inhibitors, NBCoV1 and NBCoV2, for SARS-CoV-2. We selected the SARS-CoV-2 S protein trimer in a prefusion state, as we hypothesize that these inhibitors bind to this trimer to prevent the formation of 6-HB, which is necessary for viral fusion with cells. We also tested any possible binding of these inhibitors with the SARS-CoV-2 S1 subdomain, which contains the RBD that binds the ACE2 receptors expressed on the host cell. SPR can be used to measure binding constant (K_D_) as well as k_on_ (also known as the association constant, k_a_) and k_off_ (also known as the dissociation constant, k_d_). Small-molecule inhibitors were passed through the chip surface, and the signal changes (in AU) of each inhibitor, tested at various concentrations, were recorded (Figure 3a–d). The resulting data were fit to a 1:1 binding model. The binding affinity, K_D_, and kinetic parameters, k_on_ and k_off_, for the target protein interactions with NBCoV1 and NBCoV2 were determined (Figure 3e). The K_D_ values of NBCoV1 and NBCoV2 were 1.56 and 5.37 µM, respectively, for the SARS-CoV-2 spike trimer. However, when these inhibitors were tested against the SARS-CoV-2 S1 subdomain, the K_D_ value of NBCoV1 was approximately 5-fold higher than when bound to the prefusion S trimer. Similarly, the K_D_ value for NBCoV-2 was approximately 9-fold higher for the S1 subdomain than for the SARS-CoV-2 prefusion S trimer. These data indicate that these inhibitors most likely bind to the S2 subdomain of the SARS-CoV-2 trimer; however, the exact binding mechanism utilized by these inhibitors remains unknown. However, binding with the S2 subdomain is a reasonable possibility, as these inhibitors appear to inhibit the fusion of CoVs with the cell membrane.

### 3.7. NBCoV Small Molecules Inhibit SARS-CoV-2–Mediated Cell-to-Cell Fusion

**Figure 4:**
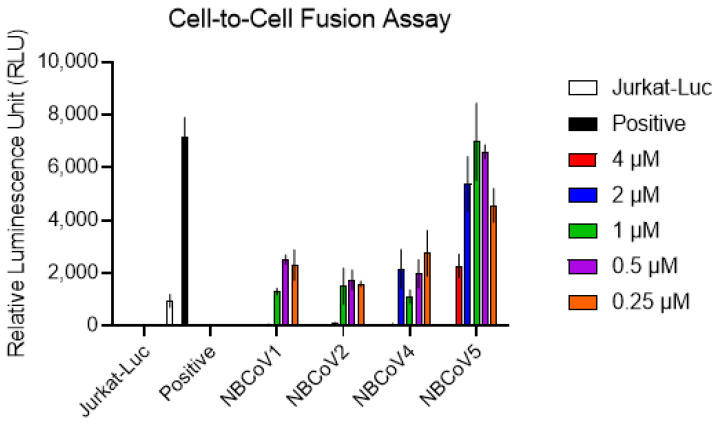
SARS-CoV-2 mediated cell-to-cell fusion inhibition assay. Jurkat cells expressing the SARS-CoV-2 full Spike wild-type gene from Wuhan-Hu-1 isolate and the luciferase gene were used as donor cells and the 293T/ACE2 as acceptor cells. Jurkat cells were preincubated for 1 h with different concentrations of NBCoV small molecules. Positive represent 293T/ACE2 cells cocultured with Jurkat cells in the absence of NBCoVs. Jurkat-Luc represents 293T/ACE2 cells cocultured with Jurkat cells expressing the luciferase gene only, in the absence of NBCoVs. Columns represent the means ± standard deviations (*n* = 2–4).

Efficient virus spread can be achieved through either a cell-free or a cell-associated mode, which involves direct cell-to-cell contacts and fusion [73]. Cell-to-cell fusion transmission permits the virus to infect adjacent cells without producing free virus particles, contributing to tissue damage and inducing syncytia formation. The interaction between ACE2 and the SARS-CoV-2 S protein and subsequent conformational changes that occur in the S protein are critical for initiating the fusion of infected cell membranes with those of adjacent cells [74,75]. Our data suggest that NBCoV compounds can inhibit SARS-CoV-2 by binding with the SARS-CoV-2 prefusion S trimer; therefore, we investigated whether our best compounds in pseudovirus inhibition assays, NBCoV1, NBCoV2, and NBCoV4, could prevent SARS-CoV-2–mediated cell-to-cell fusion. NBCoV5 was used as a negative control because we found no meaningful anti–SARS-CoV-2 activity when using this compound. We have established a novel cell-to-cell fusion assay in our laboratory, utilizing Jurkat cells expressing the luciferase gene and SARS-CoV-2 WT S protein, as donor cells and 293T/ACE2 cells as acceptor cells. We chose Jurkat cells because they grow in suspension, and unfused cells can easily be removed from wells with two PBS washes. We found that NBCoV5 only inhibited SARS-CoV-2–mediated cell-to-cell fusion at higher doses (68% inhibition at 4 µM; Figure 4), whereas NBCoV1, NBCoV2, and NBCoV4 potently inhibited cell-to-cell fusion, even at the lowest dose used in this assay (250 nM), which was associated with 62–79% inhibition of SARS-CoV-2–mediated cell-to-cell fusion. These data suggest that the binding of NBCoV small molecules with SARS-CoV-2 S protein interferes with virus-mediated cell-to-cell fusion.

### 3.8. In Vitro ADME Assessment

The in vitro assessment of ADME properties during the early stages of drug discovery and development, particularly in the pharmaceutical industry, has significantly reduced the drug attrition rate over the last two decades [76]. In 1997, the major cause of drug failure, preventing advancement to clinical trials, was poor ADME properties [77]. Drug development failures that occur during the later stages of drug discovery can be very costly. Therefore, we also performed in vitro ADME assessments of one of our potent pan-CoV inhibitors, NBCoV1, which features potent antiviral activity, low cytotoxicity, and excellent SI, to determine whether these fusion inhibitors have potential as preclinical drug candidates.

Solubility represents a key drug-like property and plays a critical role in drug discovery. Therefore, we measured the solubility of NBCoV1 in phosphate buffer at pH 7.4 (Table 7), which was low. However, solubility can be improved through salt formation or formulations. Due to the presence of a COOH anion in all potent NBCoV inhibitors, sodium, calcium, and potassium salts can be generated to enhance the solubility and dissolution rate [78,79]. We next measured NBCoV1 permeability, which contributes to drug absorption in the intestine, affecting bioavailability. Compounds with low permeability may be less well absorbed, showing poor bioavailability. The human epithelial cell line Caco-2 is the most widely used cell line for measuring permeability, simulating human intestinal absorption. Therefore, we performed a Caco-2 bidirectional permeability experiment (apical to basolateral (A–B) and basolateral to apical (B–A) across a Caco-2 cell monolayer), which can be used to measure the efflux ratio and predict the human intestinal permeability of orally administered drugs. The data shown in Table 7 indicates that the apparent permeability (P_app_) of NBCoV1 was similar to that of the orally administered drug propranolol (19.7 × 10^−6^ cm/s; See Supporting Materials). We used valspodar, a P-glycoprotein (P-gp) substrate, as a positive control to determine whether active efflux mediated by P-gp was involved in NBCoV1 permeability. After treatment with 1 µM valspodar, the efflux ratio did not change compared with cells without valspodar treatment, indicating that the P-gp–mediated efflux mechanism was not involved in NBCoV1 absorption.

Next, we examined the metabolic stability of NBCoV1 in the human liver microsome because the liver represents the most crucial site of drug metabolism in the body. The clearance interval (Cl_int_; Table 7) indicated that NBCoV1 is a low-clearance compound, with a half-life (t_1/2_) of 112 min. Low clearance rates are often targeted by drug discovery projects because low clearance rates allow for a reduction in drug dosing, minimizing the whole-body exposure to drugs, reducing drug-related toxicities, and prolonging the t_1/2_ value of the drug. Compounds with high clearance values may be cleared rapidly from the body, and drugs with short durations of action may require multiple doses. We also measured the binding of NBCoV1 in human plasma, which revealed > 99.5% was bound to plasm proteins (Table 7). It is noteworthy that many drugs show >98% plasma protein binding, which does not affect the success of drug candidates. The misconception that high plasma protein binding has effects on drug activity was elegantly refuted by Smith et al. in 2010 [80].

The cytochrome P450 (CYP450) enzyme family plays a critical role in the oxidative biotransformation of many drugs and other lipophilic xenobiotics into their hydrophilic counterparts, facilitating elimination from the body [81,82]. Greater than 50 CYP450 enzymes have been identified as belonging to the CYP450 family, although only some have been identified to have essential roles in drug metabolism, including CYP1A2, CYP2B6, CYP2C8, CYP2C9, CYP2C19, CYP2D6, CYP3A4, and CYP3A5, which are involved in the metabolism of nearly 80% of all known drugs [83,84]. Therefore, we decided to use this set of eight CYP450 enzymes to determine whether NBCoV1 has inhibitory effects on this subfamily of enzymes, which may cause potential drug–drug interactions (DDI) when co-administered with other treatment agents. DDI is a potential concern for both the pharmaceutical companies developing drugs and regulatory agencies, such as the FDA.

The following guidelines are used for assessing CYP inhibition [85]:

IC_50_ > 10 µM (CYP inhibition low)

<10 µM (CYP inhibition moderate)

<3 µM (CYP inhibition high)

Based on the above classification, NBCoV1 showed low-level inhibition against CYP2D6 and CYP3A, moderate inhibition against CYP1A2, CYP2B6, CYP2C9, and CYP2C19, and high-level inhibition against only CYP2C8 (Table 7).

### 3.9. In Vivo Pharmacokinetics of NBCoV1 and NBCoV2

Successful drug discovery depends not only on the preclinical efficacy and toxicity profile of a compound but also on the selection of the right candidate with good in vivo PK characteristics in animals (rats, dog, etc.) using appropriate dosing routes, such as oral (PO) and intravenous (IV) administration.

We evaluated the PK parameters of two of the most active inhibitors in rats (Table 8) following administration by PO and IV routes. The t_1/2_ following the PO administration of NYBCoV1 was 11.3 h, whereas that for IV administration was 3.57 h. NBCoV1 dosed via IV showed a time to reach the maximum concentration (T_max_) of 0.25 h, compared with 2 h when administered PO, suggesting normal clearance. The maximum measured plasma concentration (C_max_) values for NBCoV1 and NBCoV2 were 1499 ng/mL and 2219 ng/mL, respectively. NBCoV1 also showed an excellent mean residence time (MRT) of 14 h, which represents the average time a drug molecule spends in the body and is critically important for a drug to elicit its action. The oral bioavailability of NBCoV1 was reasonably good (F% = 20%) for initiating further preclinical studies. However, the bioavailability of NBCoV1 can be further improved through proper dosing, salt formation, or proper clinical formulation. NBCoV2 showed poor oral availability, with an F% of 0.9%, and the PO and IV administration t_1/2_ values ranged from 3.5 to 3.9 h. NBCoV2 dosed via IV showed a T_max_ of 2 h, suggesting that the compound may precipitate following injection and require redissolution, delaying maximum blood levels, suggesting that PK studies should be examined using even lower doses (1–3 mg/kg body weight).

### 3.10. Confirmation That NBCoVs Are Not Promiscuous Binders or Aggregators

#### 3.10.1. The Antiviral Activities of Ene-Rhodanine Derivatives Are Not Due to Luciferase Activity Inhibition or Direct Interference with Luminescence Measurements

Initial identification of antiviral activity was performed against a lentiviral-based pseudotyped virus containing the SARS-CoV-2 S protein using a NanoLuc (luciferase-based) assay. One possibility was that ene-rhodanines might directly inhibit the luciferase enzyme; however, of the 13 compounds tested, only 3 or 4 showed antiviral potency, despite all compounds containing the same ene-rhodanine scaffold. In virus neutralization assays, we pretreated the pseudoviruses with the small molecules for 30 min before cell infection because the target of our study was the SARS-CoV-2 S protein. By contrast, when we pretreated the cells rather than the viruses, no inhibition was observed, even at the highest doses used in the assay (2000 nM; Table S1). These experiments suggested that (1) our compounds do not affect NanoLuc activity; and (2) the target of these compounds is virus-related and not cell related. In 2008, Auld et al. reported the luciferase inhibitory activity of >72,000 diverse molecules collected from a diverse chemical repository. Twenty-six rhodanines were also tested against the luciferase enzyme, and none showed any inhibitory activity [86]. In this work, to explicitly exclude the possibility that the antiviral activity associated with our small molecules was due to the direct inhibition of the NanoLuc and the FLuc reporters, we expressed these enzymes in 293T/17 cells. We incubated the cells lysates with 2000 nM of NBCoV small molecules (4 small molecules with the highest inhibitory activities, NBCoV1–4, and two inactive compounds, NBCoV5 and NBCoV34) for 10 min at 25 °C. As controls, lysates were treated with and without Intracellular TE Nano-Glo^®^ Substrate/Inhibitor (for the NanoLuc reporter) and 100 µM resveratrol [87] (inhibitor of the FLuc reporter). We found that while the NanoLuc inhibitor (Figure S4a) and the FLuc inhibitor (Figure S4b) completely blocked the activities of their respective luciferase enzymes, the NBCoV compounds did not affect the activities of these two enzymes (Figure S4a,b). No significant differences were detected between the untreated controls and the samples treated with the NBCoV small molecules.

The second concern is that rhodanines might interfere with the luminescence-based measurements. However, we used the same luciferase enzymes in control experiments using A-MLV–based pseudovirus when we performed the cell–cell fusion assay. However, none of the rhodanine derivatives were found to be active against the A-MLV pseudovirus. By contrast, the most active leads in the SARS-CoV-2 pseudovirus assay also showed activity in the cell–cell fusion assay. These findings indicate that the observed activities are specific to SARS-CoV-2 and are not due to non-specific interference with luminescence-based measurements.

The most intriguing argument can be made based on the assay results using the authentic SARS-CoV-2 virus, which did not utilize a luciferase reporter. The cells (Vero) used in this assay also differed from those used in a pseudovirus inhibition assay. In addition, during the authentic virus assay, a microscope was used to determine the CPE, which represents a completely different readout. When the cells (rather than the virus) were pretreated for 2 h before infection, no anti–SARS-CoV-2 protection was observed for even the highest tested dose (10 µM; Table S1). These concurrent assays firmly establish that outcomes associated with rhodanine administration were specific for the SARS-CoV-2 S protein.

#### 3.10.2. Target Specificity Measured by a Direct Binding Study by SPR

As suggested by the ACS panel [88], we went a step further by measuring the direct binding of the most active inhibitors with the target SARS-CoV-2 S protein using SPR. We hypothesized that these inhibitors bind the HR1 domain of the S protein, preventing 6-HB formation, similar to the mode of inhibition observed for HIV-1 or other Class I fusion proteins expressed by enveloped viruses. We examined the binding of NBCoV small molecules with the SARS-CoV-2 prefusion S trimer and observed low-µM K_D_ values for NBCoV1 and NBCoV2. Although these inhibitors showed some binding with the S1 domain, the K_D_ values were 5–9-fold higher than for the S2 domain. Therefore, this critical study demonstrated that these inhibitors preferentially bound with the prefusion formation of the S protein, supporting our hypothesis. We do not know the exact binding location for NBCoVs; however, future X-ray or cryo-electron microscopy structural determination can be performed for these inhibitors, providing information regarding the exact binding sites.

#### 3.10.3. The Inhibitors Specifically Bind the Viral S Protein

In 2014, Baell and Walters, in their comments published in Nature, referred to ene-rhodanines as among the “most insidious” offenders” [89], characterized by promiscuous protein binding. They also mentioned that this scaffold primarily acts through covalent modifications and the formation of metal complexes. We do not know whether our inhibitors participate through any such mechanisms without structural information. However, we present some experimental evidence indicating that the inhibitors bind to the virus, not the cellular components, which most likely does not involve the ene-rhodanine scaffold. To accomplish our goal, we used a two-pronged approach.

(1). The timing of compound addition is critical for antiviral activity.

In the pseudovirus-based inhibition assay, the entry/fusion inhibitors targeting the viral envelope/S proteins do not show inhibitory effects if added to the cell prior to virus addition. We successfully demonstrated that when we incubated the compounds with the cells first, followed by pseudovirus addition, none of the compounds showed inhibitory activities (Table S1). However, if we reversed the sequence by pre-incubating the virus with the compound before adding them to the cells, the compounds showed dose–response inhibition (Table 1, Table 2, Table 3 and Table 6).

In the authentic, live SARS-CoV-2 virus inhibition assay, we demonstrated a similar effect (Table 5), in which none of the tested compounds showed any inhibitory activity when first added to the cells (Table S1).

These experiments conclusively established that the inhibitors were targeting the virus itself rather than any cellular components.

(2). Cellular toxicity vs. antiviral activity.

During the pseudovirus-based inhibition assay, cellular toxicity was assessed for each compound in the absence of viral infection. The data presented in Table 1 and Table 3 indicate that all active compounds have low cytotoxicity, resulting in SI values that ranged from >586 to 4000. If the compounds targeted a cellular component, then the SI values would have been much lower. Based on the ACS panel’s recommendations, we demonstrated that “the compound is active at a concentration substantially lower than those producing cellular toxicity” [88].

#### 3.10.4. The NBCoV Small Molecules Are Not Promiscuous Aggregation-Based Inhibitors

Based on the steps outlined by the ACS editor panel for excluding inhibitory activity due to colloidal aggregation [88], we investigated these compounds further. One suggestion was the use of publicly available filters; therefore, we used the online software Advisor, which was developed by Shoichet’s team at UCSF (http://advisor.bkslab.org/; accessed on 17 June 2021). The software returned with a message that none of the compounds were like any known aggregators in their database (Supporting Materials, Figure S5). However, the software also suggested that because the molecules are hydrophobic, other appropriate tests should be performed. The authors suggested that if the activity of an aggregation-based inhibitor can be attenuated by small concentrations of nonionic detergent (0.025% Tween-80), the compound is likely to be acting as an aggregator [90,91]. A colloidal aggregator typically exhibits a steep dose–response curve and may be precipitated by centrifugation [92,93,94,95]. Due to the different sensitivities of cells to detergents, we initially performed a cytotoxicity assay using 293T/ACE2 cells in the presence and absence of 0.025% of Tween-80. Unfortunately, we found that even low concentrations of Tween-80 induced significant cytotoxicity (Figure S6a). Moreover, in the presence of 0.025% Tween-80, the infection of 293T/ACE2 cells with the SARS-CoV-2 pseudovirus dramatically decreased (Figure S6b) compared with infection performed in the absence of Tween-80, suggesting that 0.025% of Tween-80 may affect both viral and cell viability. We then performed the viral neutralization assay using NBCoV1 supernatant, following centrifugation to eliminate ‘eventual’ colloidal aggregates. The dose–response results obtained using the centrifuged NBCoV1 were very similar to those obtained with uncentrifuged NBCoV1 (NBCoV1-control; Figure S6c). No significant difference in the calculated IC_50_ values was observed, which were 50 nM for NBCoV1-control and 45 nM for NBCoV1-centrifuged, suggesting that NBCoV1 viral inhibitory activity was not due to colloidal aggregation.

Additionally, in 2003, Seidler et al. [96] suggested that potential aggregators can be screened for the inhibition of three unrelated enzymes, specifically, β-lactamase, trypsin, and malate dehydrogenase (MDH), which are highly sensitive to compound aggregation. One of their criteria suggested that a compound can be considered promiscuous if the compound inhibits all three enzymes. We evaluated the activities of 6 NBCoV compounds (4 compounds with the highest inhibitory activities, NBCoV1–4, and two inactive compounds, NBCoV5 and NBCoV34) at 2000 nM against these three enzymes using a colorimetric assay. As shown in Table S2, we found that the tested NBCoV compounds had no inhibitory activities against β-lactamase, trypsin, or MDH, indicating that these compounds should not be considered aggregators.

Therefore, we demonstrated through a series of rationale and controlled experiments, according to the recommendation of the ACS editor panel and others [88], that the pan-CoV inhibitors presented in this article genuinely target the viral component, specifically the S protein, to elicit true antiviral potency.

## 4. Discussion

The current COVID-19 pandemic has reiterated the importance of continued focus on the discovery of potent, non-toxic small-molecule pan-CoV drugs that are orally available and affordable, allowing these drugs to treat billions of people worldwide to rapidly counter this type of pandemic in the future. Despite the rapid development of several vaccines against SARS-CoV-2, millions of people are refusing to take the vaccine out of fear of insufficient due diligence. Furthermore, many reports have described breakthrough infections and deaths, even after double vaccination, although other comorbidity factors may have contributed to those deaths. Due to the urgent need to discover new anti-CoV drugs, we utilized our long experience with the development of HIV-1 fusion inhibitors because both HIV and CoVs use similar fusion mechanisms, as detailed in the introduction.

We repurposed some earlier discovered HIV-1 gp41-targeted fusion inhibitors to screen against the three most critical CoVs identified in humans thus far: SARS-CoV, SARS-CoV-2, and MERS-CoV. Peptide-based pan-CoV inhibitors have been reported with promising antiviral potency. However, peptide-based drugs, especially for large-scale use, may not be cost-effective. We successfully identified several small-scale aromatic/heteroaromatic carboxylic acid–based inhibitors (NBCoV1–NBCoV4) that potently inhibited all three CoVs (IC_50_ values < 200 nM) with very low cytotoxicity and remarkable SI. The acid group in the meta position of the phenyl ring is critical, as are the hydrophobic groups connected to the ene-rhodanine moiety [41]. We observed a similar SAR as that observed for the HIV-1 gp41-based fusion inhibitors. NBCoV15 has the same hydrophobic group attached to the ene-rhodanine moiety but lacks the COOH group in the meta position, showing virtually no inhibition. Similarly, NBCoV17 has identical substituents on the phenyl ring, located at the same positions, but lacks the hydrophobic group attached to the ene-rhodanine moiety and shows no antiviral activity.

Since the publications regarding PAINS, by Baell and Holloway [97], and colloidal aggregators, by McGovern et al., which revealed that some substructures are often frequent hitters or promiscuous inhibitors [98], based on high-throughput screening assays, increased awareness of these categories of compounds have become important during drug discovery. However, several publications have argued that not all frequent hitters should be randomly discarded without first validating whether they are target-specific or truly promiscuous [58,99,100,101,102]. In a recent editorial, Bajorath mentioned that the chemical integrity and specific biological activity of compounds containing PAINS substructures should be considered in the context of the whole compound and how they are embedded in the structure. He also argued that “PAINS-induced activity artifacts cannot be generalized but require careful assessment on a case-by-case basis” [99]. Based on the legitimate concerns regarding PAINS and colloidal aggregates, nine American Chemical Society (ACS) editors have outlined the necessary steps that must be performed to exclude artifactual assay activities [88]. The goal of this concerted effort is “not to eliminate a priori all compounds that may resemble PAINS or colloidal aggregators” but to ensure that the compounds’ “behavior is well-vetted before publication”.

Ene-rhodanines have been designated as frequent hitters, and the most likely activities associated with this series of compounds are speculated to be independent of the actual target. By contrast, Mendgen et al., in 2012, conclusively demonstrated that rhodanines and thiohydantoins possess distinct molecular interaction patterns that are governed by their electronic and hydrogen bonding properties rather than due to promiscuous binding or aggregation. Therefore, the authors suggested that these scaffolds should not be viewed as problematic or promiscuous binders [58]. We respect both views; thus, in the spirit of the suggestions made by the ACS editor panel [88] and others [99,103], we validated the antiviral activities of the set of rhodanines that we have presented here.

The SAR above clearly demonstrate that despite the presence of ene-rhodamine, some of these molecules showed no anti-CoV activity. This study further validates that mere presence of those moieties does not make a drug promiscuous. We demonstrated through multiple controlled experiments that the NBCoV series of pan-CoV inhibitors are not promiscuous or aggregators.

The antiviral potencies (IC_50_ values) of NBCoV-1–NBCoV4 were measured in the low-nM range. However, all the compounds consistently displayed better activity when tested in 293T/ACE2 and HT1080/ACE2 cells than when tested in A549/ACE2 cells. Also, in Vero cells, NBCoV1–4 appeared to be less potent against the authentic SARS-CoV-2 virus, using the complete inhibition of CPE (IC_100_) as endpoint (1.25 to 2.5 µM), than against the pseudovirus (IC_100_ from 0.25 to 1.2 µM). Variation of antiviral activity in different cell lines has been previously described [67]. A similar observation was reported for remdesivir, a drug that has received FDA emergency use authorized for SARS-CoV-2 treatment, in an NLuc-based assay [104]. In A549-hACE2 cells, remdesivir was >10-fold more active (115 nM) than when tested in Vero E6 cells (1.28 µM). Although those two assays could not be directly compared, there appears to be a difference in the antiviral potency between different cell lines.

In a recent study it has been shown that TMPRSS2 expression influences the entry routine used by SARS-CoV-2 [61]. In our studies, together with the low toxicity, exposing the cells to the NBCoVs before infection does not prevent infection, suggesting that these compounds per se do not affect the cells. Additionally, we could not find a direct correlation between expression levels of TMPRSS2 and potency of the inhibitors (Figures S2 and S3) therefore, more investigation will be necessary. All four of the most active inhibitors in the pseudovirus assay also demonstrated high sensitivity against laboratory-generated mutants that mimic existing VOCs. For the first time, a variant (Delta, a member of the B.1.617 lineage) with a mutation in the S2 domain (D950N) of the SARS-CoV-2 S protein was identified [72]. Because we hypothesized that our COOH-containing molecules might interfere with the formation of the salt bridge between K947 and D1182 in the S protein, we were concerned that the D950N mutation might have detrimental effects on the inhibitory activities of NBCoV compounds. However, a close inspection of the postfusion domain structure of the S protein revealed that despite proximity in amino acid sequence, residue 950 is located far from the critical K947 residue (data not shown). We also found that two of the most active inhibitors, NBCoV1 and NBCoV2, inhibited these mutants. In short, the most active inhibitors also showed their great potential to be useful against COVID-19 variants.

Although we demonstrated the binding of NBCoV1 with the prefusion domain of the SARS-CoV-2 S protein, we have not yet confirmed the specific binding location of these S protein inhibitors. We are currently undertaking cryo-electron microscopy–based structural determinations of the binding status of these inhibitors with the S protein during the prefusion state, which is expected to identify the binding site and ascertain the mechanism of action.

## 5. Conclusions

Based on the remarkable similarity in the fusion mechanism between the CoV S protein and envelope glycoproteins in HIV-1, we discovered a series of pan-CoV fusion inhibitors, which also show potent inhibition against recently identified COVID-19 variants, including those identified in the UK (Alpha), South Africa (Beta), and India (Delta). Among 13 tested compounds tested, we found at least three that showed low-nM IC_50_ values during a pseudovirus-based inhibition assay. These molecules also showed the complete inhibition of CPE (IC_100_) against an authentic, live virus, SARS-CoV-2 (US_WA-1/2020), tested in Vero cells. Although limited, the SAR indicates that a balance of electrostatic and hydrophobic interactions is required for optimal antiviral activity. For example, when a phenylethyl moiety was replaced by an H or a smaller hydrophobic group, the inhibitory activities of those compounds disappeared. The SAR also showed room for the further derivatization of the phenylethyl moiety. A direct binding study using SPR confirmed that these molecules bind to the prefusion trimer of the SARS-CoV-2 S protein more tightly than they bind the S1 subdomain. A subsequent cell-to-cell fusion assay confirmed that these inhibitors efficiently prevent virus-mediated cell-to-cell fusion. We also demonstrated, through a series of rationally designed experiments, that these inhibitors are not promiscuous but are true pan-CoV inhibitors, despite the presence of an ene-rhodanine scaffold, which has been defined by some as being associated with “frequent hitters”. As part of our early drug discovery protocol, we also performed an ADME study, which indicated that the solubility of these inhibitors requires further improvements, either through chemical modifications or through salt formation. All other measured ADME properties showed drug-like characteristics. Furthermore, the PK study in rats demonstrated that NBCoV1 has all the desirable features, including 20% oral availability, for consideration in further preclinical assessments. Overall, we discovered a set of novel, small-molecule pan-CoV fusion inhibitors that are likely treatment candidates with great potential to be developed into therapeutic agents for COVID-19 and related CoV diseases.

## Figures and Tables

**Table 1:** Antiviral activity of the NBCoV small molecules in the single-cycle assay in different cell lines against pseudovirus NL4-3ΔEnv-NanoLuc/SARS-CoV-2 (IC_50_), toxicity (CC_50_), and selectivity index (SI).

Compound	293T/ACE2 Cells			HT1080/ACE2 Cells			A549/ACE2 Cells		
	IC_50_ (nM) ^a^	CC_50_ (µM) ^a^	SI	IC_50_ (nM) ^a^	CC_50_ (µM) ^a^	SI	IC_50_ (nM) ^a^	CC_50_ (µM) ^a^	SI
NBCoV1	51 ± 17	38.5 ± 1	755	32.3 ± 4.6	89 ± 2	2755	63.6 ± 4.6	86 ± 8.7	1352
NBCoV2	22.8 ± 0.8	37.5 ± 2	1630	25.3 ± 0.6	>100	>4000	58 ± 1.7	>100	>1724
NBCoV3	60.1 ± 8.5	45 ± 0.5	750	64 ± 18	>100	>1563	120 ± 5	>100	>833
NBCoV4	26 ± 1	40.7 ± 2.3	1565	47.7 ± 16	>100	>2096	73 ± 4.1	>100	>1370
NBCoV5	1205 ± 240	35 ± 2	29	1050 ± 252	>100	>95	>2000	>100	N/A ^b^
NBCoV6	185 ± 5.8	40 ± 2.6	216	245 ± 5	>100	>408	613 ± 72	>100	>163
NBCoV7	298 ± 60	45 ± 0.4	151	290 ± 57	>100	>345	416 ± 25	>100	>240
NBCoV8	65.8 ± 6.2	38.7 ± 1.2	586	94 ± 9	>100	>1063	254 ± 27	>100	>394
NBCoV9	342 ± 46	33.7 ± 2.5	98	365 ± 73	>100	>274	596 ± 42	>100	>168
NBCoV15	>2000	64 ± 14	N/A ^b^	>2000	>100	N/A ^b^	N/A ^b^	N/A ^b^	N/A ^b^
NBCoV17	>2000	>100	N/A ^b^	>2000	>100	N/A ^b^	N/A ^b^	N/A ^b^	N/A ^b^
NBCoV28	>2000	>100	N/A ^b^	>2000	92 ± 3	N/A ^b^	N/A ^b^	N/A ^b^	N/A ^b^
NBCoV34	>2000	83 ± 4	N/A ^b^	>2000	~100	N/A ^b^	N/A ^b^	N/A ^b^	N/A ^b^

^a^ The reported IC_50_ and CC_50_ values represent the means ± standard deviations (*n* = 3). ^b^ Not Available.

**Table 2:** Antiviral activity of the NBCoV small molecules in a single-cycle assay in different cell lines against pseudovirus NL4-3ΔEnv-NanoLuc/SARS-CoV (IC_50_) and selectivity index (SI).

Compound	293T/ACE2 Cells		HT1080/ACE2 Cells		A549/ACE2 Cells	
	IC_50_ (nM) ^a^	SI	IC_50_ (nM) ^a^	SI	IC_50_ (nM) ^a^	SI
NBCoV1	17 ± 2.6	2265	39 ± 8.2	2282	98 ± 4	878
NBCoV2	13.8 ± 0.2	2717	19.3 ± 1.1	>5181	111 ± 9	>901
NBCoV3	17.8 ± 4	2528	25.7 ± 0.6	>3891	157 ± 12.5	>637
NBCoV4	80 ± 2	509	54 ± 3.5	>1852	100 ± 13	>1000
NBCoV5	1853 ± 179	19	1760 ± 330	>57	>2000	N/A ^b^
NBCoV6	175 ± 7	229	133 ± 9.5	>752	867 ± 62	>115
NBCoV7	111 ± 1.7	405	52 ± 8.9	>1923	271 ± 76	>369
NBCoV8	128 ± 6.7	302	225 ± 26	>444	700 ± 70	>143
NBCoV9	217 ± 22	155	246 ± 10	>407	350 ± 15	>286

^a^ The reported IC_50_ values represent the means ± standard deviations (*n* = 3). ^b^ Not Available.

**Table 3:** Antiviral activity of the NBCoV small molecules in a single-cycle assay in different cell lines against pseudovirus NL4-3ΔEnv-NanoLuc/MERS-CoV (IC_50_), toxicity (CC_50_), and selectivity index (SI).

Compound	HuH-7 Cells			MRC-5 Cells		
	IC_50_ (nM) ^a^	CC_50_ (µM) ^a^	SI	IC_50_ (nM) ^a^	CC_50_ (µM) ^a^	SI
NBCoV1	95 ± 22	~100	1053	76.5 ± 0.3	69 ± 1	902
NBCoV2	112 ± 7	80 ± 1	714	77. ± 0.5	63 ± 2.8	818
NBCoV3	158 ± 14	92 ± 2	582	123 ± 17	>50	>407
NBCoV4	131 ± 6	80 ± 6	611	80 ± 18	63.5 ± 3.5	794
NBCoV5	>2000	76 ± 2	N/A ^b^	>2000	72.5 ± 1	N/A ^b^
NBCoV6	569 ± 31	71 ± 1	125	945 ± 77	71 ± 2.8	75
NBCoV7	933 ± 153	N/A^b^	N/A ^b^	927 ± 63.5	68.8 ± 1	74
NBCoV8	509 ± 43	83 ± 2	163	559 ± 66	67.5 ± 3.5	121
NBCoV9	214 ± 23	55 ± 9	257	232 ± 17	42.5 ± 3.5	183

^a^ The reported IC_50_ and CC_50_ values represent the means ± standard deviations (*n* = 3). ^b^ Not Available.

**Table 4:** Antiviral activity of the NBCoV small molecules against control pseudovirus NL4-3.Luc.R-E-/A-MLV (IC_50_) evaluated in 293T/ACE2 cells.

Compound	293T-ACE2 Cells/ A-MLV
	IC_50_ (nM) ^a^
NBCoV1	1397 ± 12
NBCoV2	783 ± 76
NBCoV3	960 ± 57
NBCoV4	1623 ± 115
NBCoV5	>2000
NBCoV6	>2000
NBCoV7	>2000
NBCoV8	>2000
NBCoV9	1627 ± 118

^a^ The reported IC_50_ values represent the means ± standard deviations (*n* = 3).

**Table 5:** Antiviral activity (IC_100_) of the NBCoV small molecules in Vero E6 cells infected with SARS-CoV-2 (US_WA-1/2020) and the range of IC_100_ detected in the three cell lines 293T/ACE2, HT1080/ACE2, and A549/ACE2 infected with NL4-3ΔEnv-NanoLuc/SARS-CoV-2.

Compound	SARS-CoV-2 (US_WA-1/2020)	NL4-3ΔEnv-NanoLuc/SARS-CoV-2
	Vero E6 Cells	293T/ACE2, HT1080/ACE2 and A549/ACE2 Cells
	IC_100_ (µM) ^a^	IC_100_ (µM) ^b^
NBCov1	1.25	0.25–1
NBCoV2	1.25	0.3–1
NBCoV3	2.5	0.5–1.2
NBCoV4	2.5	0.3–1
NBCoV5	>10	>2
NBCoV6	>10	1–3
NBCoV7	5	1–3
NBCoV8	5	1–2.5
NBCoV9	2.5	2–3

**^a^** Values indicate the lowest concentration capable of completely preventing virus-induced CPE in 100% of the wells. **^b^** Values indicate the range of IC_100_ values detected in the three cell lines.

**Table 6:** Antiviral activity of the NBCoV small molecules against NL4-3∆Env-NanoLuc/SARS-CoV-2 mutant pseudoviruses variants B.1.1.7 UK (Alpha), B.1.351 RSA (Beta) and B.1.617.2 India (Delta).

Compound →	NBCoV1 IC_50_ (nM) ^a^	NBCoV2 IC_50_ (nM) ^a^	NBCoV3 IC_50_ (nM) ^a^	NBCoV4 IC_50_ (nM) ^a^	NBCoV5 IC_50_ (nM) ^a^
SARS-CoV-2 ↓					
**WT**	51 ± 17	22.8 ± 0.8	60.1 ± 8.5	26 ± 1	1205 ± 240
**B.1.1.7 UK (Alpha)**					
N501Y	52.5 ± 0.2	49 ± 0.5	44.5 ± 0.5	51 ± 0.4	>2000
∆69–70	48 ± 3	55 ± 0.5	69 ± 7	53 ± 0.5	>2000
P681H	35 ± 0.5	51 ± 0.3	55 ± 0.3	44 ± 0.4	1720 ± 215
N501Y/∆69–70	46 ± 0.5	66 ± 1	94 ± 16	53 ± 11	>2000
N501Y/∆69–70/P681H	57 ± 0.5	79 ± 14	232 ± 17	158 ± 31	>2000
**B.1.351 RSA (Beta)**					
E484K	42 ± 0.7	45 ± 0.2	80 ± 10.6	45 ± 0.4	>2000
D614G	56 ± 0.6	61 ± 0.3	145 ± 28	58 ± 0.4	1730 ± 62.5
N501Y/D614G	57 ± 2	59 ± 13	104 ± 1	77 ± 29	>2000
E484K/N501Y	32 ± 0.5	36 ± 0.4	42 ± 1	51 ± 5	>2000
E484K/D614G	33 ± 0.5	35 ± 0.2	44 ± 0.8	44 ± 3	1846 ± 124
E484K/N501Y/D614G	45 ± 2	43 ± 0.5	101 ± 2	43 ± 2.5	>2000
**B.1.617.2 India (Delta)**					
D950N	47.7 ± 15	88.7 ± 6	N/A ^b^	207 ± 29	>2000
D614G/P681R	49 ± 13	65 ± 18	N/A ^b^	148 ± 30	>2000
D614G/D950N	33 ± 10	26 ± 2	N/A ^b^	77.5 ± 16.5	>2000
D614G/P681R/D950N	58 ± 7	53 ± 12	N/A ^b^	239 ± 7	>2000

^a^ The reported IC_50_ values represent the means ± standard deviations (*n* = 3). ^b^ Not Available.

**Table 7:** In vitro ADME profile of one of the most potent inhibitors NBCoV1.

Assay Performed		In Vitro ADMET	Inhibitor
			NBCoV1
Solubility (µM)		Phosphate buffer, pH7.4	4.72
Caco-2 permeability (mean P_app_, × 10^−6^ cm/s)	NBCoV1	A-to-B	16.9
		B-to-A	20.4
		Efflux Ratio	1.21
	NBCoV1 + 1 µM valspodar	A-to-B	20.5
		B-to-A	23.6
		Efflux Ration	1.15
	P-gp Substrate classification	-	Negative
Metabolic Stability (human liver microsomes)		Cl_int_ (mL/min/mg protein)	0.0124
		Half-life (min)	112
Protein binding (human plasma)		% bound	>99.5
Cytochrome P450 inhibition, IC_50_ (µM)		CYP1A2 (Phenacetin)	7.40
		CYP2B6 (Bupropion)	3.19
		CYP2C8 (Amodiaquine)	2.08
		CYP2C9 (Diclofenac)	5.01
		CYP2C19 (S-Mephenytoin)	7.31
		CYP2D6 (Bufuralol)	>10
		CYP3A (Midazolam)	>10
		CYP3A (Testosterone)	>10

**Table 8:** In vivo PK parameters in rats of the two most active inhibitors, NBCoV1 and NBCoV2.

Parameters ^a^	NBCoV1		NBCoV2		UNITS
	PO	IV	PO	IV	
Dose	10	5	10	5	mg/kg
t_1/2_	11.32	3.57	3.96	3.52	h
T_max_	2	0.25	4	2	h
C_max_	1499.76	7815.76	30.12	2219.45	ng/mL
C_0_	-	7407.25	-	1710.87	ng/mL
AUC_0-t_	12,023.00	37,515.91	318.78	18,074.45	ng/mL ∗ h
MRT_0-inf_obs_	14.34	4.02	7.28	5.09	h
CL_obs_	-	0.00013	-	0.00027	(mg/kg)/(ng/mL)/h
Vss__obs_	-	0.00053	-	0.00140	(mg/kg)/(ng/mL)
Vz/F__obs_	0.01078	-	0.17593	-	(mg/kg)/(ng/mL)
CL/F__obs_	0.00066	-	0.03082	-	(mg/kg)/(ng/mL)/h
F%	20.1	-	0.9	-	100 ∗ AUC(PO)/AUC(IV)

^a^ A single oral (PO) or IV dose; t_1/2_, apparent terminal elimination half-life; t_max_, time to peak concentration; C_max_, maximum measured plasma concentration; C_0_, initial measured plasma concentration; AUC, area under the concentration time curve; MRT, mean residence time; CL, clearance rate of the analyte (IV only); V_ss_, volume of distribution of the analyte in the test system estimated at steady state (IV only); VZ/F, apparent volume of distribution; CL/F, apparent oral clearance; F%, bioavailability, represents the fraction of a dose reaching systemic circulation intact; i.e., fraction of dose absorbed.

## Data Availability

Not applicable.

## Supplementary Materials

The following supporting information can be downloaded at: https://www.mdpi.com/article/10.3390/v14010069/s1, Molecular formula strings; Purification and characterization of all newly synthesized and purchased compounds (S1–S24); in vitro ADME (S25–S34); Additional Methods: Enzyme assay (S35–S36); colloidal aggregation study (S36–S37); Tables S1 and S2 (S38); Figure S1 (S39); Figure S2 (S40); Figure S3 (S41); Figure S4 (S42); Figure S5 (S43) and Figure S6 (S44).
